# Airway recanalization followed by montgomery T-tube placement for severe subglottic stenosis in an adolescent: a case report

**DOI:** 10.3389/fmed.2026.1987862

**Published:** 2026-10-28

**Authors:** Yiyi Lu, Jinbo Li, Qian Chen, Miaomiao Yuan, Kaiwang Cui, Xiaohua Lu, Gaohua Chen, Xiangwen Gong

**Affiliations:** 1 Department of Respiratory and Critical Care Medicine, Ganzhou Fifth People’s Hospital, Ganzhou Key Laboratory of Respiratory Diseases, Ganzhou Institute for Prevention and Treatment of Respiratory Diseases, Ganzhou, Jiangxi, China; 2 School of Rehabilitation Medicine, Gannan Medical University, Ganzhou, Jiangxi, China; 3 Department of Otolaryngology, Ganzhou Fifth People’s Hospital, Ganzhou Key Laboratory of Respiratory Diseases, Ganzhou Institute for Prevention and Treatment of Respiratory Diseases, Ganzhou, Jiangxi, China

**Keywords:** adolescent, airway recanalization, montgomery t-tube, pulmonary hygiene, subglottic stenosis

## Abstract

**Background:**

Post-intubation subglottic stenosis (SGS) in adolescents is a progressive acquired airway disorder that can substantially impair quality of life. Both surgical and interventional airway therapies are effective treatment options for SGS; however, each carries considerable technical demands, procedural risks, and potential complications. Interventional airway therapy relieves airway obstruction, maintains airway patency, restores airway and phonatory function, and has a less invasive profile, positioning it as a key treatment modality for SGS.

**Case presentation:**

A 15-year-old girl sustained a traumatic brain injury following a motor vehicle accident. She received endotracheal intubation and mechanical ventilation in the intensive care unit for two weeks, followed by tracheostomy. After cognitive and motor function recovered, attempted complete occlusion of the tracheostomy tube provoked dyspnea. Subsequent bronchoscopic examination revealed subglottic stenosis.

**Diagnosis:**

Bronchoscopic examination demonstrated approximately 98% subglottic airway stenosis involving a segment of roughly 2 cm with a funnel-shaped morphology, consistent with acquired post-intubation SGS.

**Interventions:**

A multidisciplinary team developed an individualized treatment strategy that included rigid bronchoscopy-assisted respiratory support, airway recanalization, Montgomery T-tube placement, perioperative pulmonary hygiene management, and surveillance for procedure-related adverse events.

**Results:**

Airway recanalization, including electrocautery incision, laser ablation, and balloon dilation, was performed successfully with rigid bronchoscopic support, restoring the tracheal lumen to approximately 1 cm in diameter. After the airway mucosa had stabilized, a Montgomery T-tube was implanted. Following T-tube placement, the patient regained phonatory function and experienced marked improvement in dyspnea. Computed tomography confirmed appropriate positioning of the Montgomery T-tube. Follow-up bronchoscopy demonstrated a patent lumen without significant secretion retention or granulation tissue formation.

## 1 Introduction

Subglottic stenosis (SGS) is characterized by abnormal narrowing of the airway between the vocal cords and the inferior border of the cricoid cartilage, resulting in airflow limitation and increased airway resistance. Acquired SGS in children and adolescents is most commonly caused by iatrogenic injury, particularly after endotracheal intubation, mechanical ventilation, and tracheostomy (1). Previous studies have reported that the incidence of post-intubation SGS in pediatric and adolescent patients admitted to the intensive care unit is approximately 11.4%, with a higher risk in patients with small body size, prolonged intubation, and repeated intubation procedures (2, 3).

**Table 1:** Clinical timeline and objective airway outcomes.

Date	Event	Key finding/outcome
Feb 2, 2026	Traumatic brain injury; endotracheal intubation	Mechanical ventilation for 2 weeks
Feb 15, 2026	Tracheostomy	Performed during severe pulmonary infection
Apr 20, 2026	Failed complete tube occlusion; airway assessment	Grade III SGS; 2-mm minimum lumen; 2-cm segment
Apr 30, 2026	Endoscopic recanalization	Lumen enlarged from ∼2 to 10 mm; immediate T-tube placement deferred
May 19, 2026	Montgomery T-tube placement	Successful after mucosal stabilization
May 22, 2026	Follow-up bronchoscopy	Tube patent and well positioned; no early granulation or secretion retention; phonation restored

Open procedures, including cricotracheal resection and laryngotracheal reconstruction, can provide definitive treatment for severe SGS but are technically demanding and may affect phonation or require prolonged recovery (4, 5). Evidence on endoscopic recanalization followed by stenting in high-grade adolescent SGS remains limited (6).

Rigid bronchoscopy, balloon dilation, scar ablation, and Montgomery T-tube placement may restore airway patency with less surgical trauma in selected patients (7). We report a staged endoscopic recanalization and T-tube strategy for severe post-intubation SGS in an adolescent.

## 2 Case presentation

A 15-year-old girl was admitted to our hospital with a history of traumatic brain injury and tracheostomy performed more than 2 months earlier. She had previously been healthy. On February 2, 2026, she was involved in a motorcycle accident that resulted in loss of consciousness. She was admitted to the neurosurgical intensive care unit and received endotracheal intubation with mechanical ventilation for 2 weeks. During hospitalization, she received supportive treatment, including sedation, analgesia, anti-infective therapy, fluid replacement, and antispasmodic therapy.

Because of severe pulmonary infection, tracheostomy was performed on February 15, 2026. Her cognitive and motor functions subsequently improved. On April 3, 2026, the tracheostomy tube was replaced with a size 8 metal tracheostomy tube. Partial tube occlusion was well tolerated; however, complete occlusion failed because of respiratory distress. Bronchoscopic examination revealed subglottic airway stenosis. The patient was aphonic; swallowing function remained normal.

On admission, her body temperature was 36 °C, pulse rate was 85 beats/min, respiratory rate was 20 breaths/min, blood pressure was 122/82 mmHg, and oxygen saturation was 97% on room air. Her height was 145 cm, and her body weight was 28 kg. A size 6 metal tracheostomy tube was in place. Auscultation revealed coarse breath sounds in both lungs, without obvious wheezing or crackles. No significant abnormalities were identified on cardiovascular or abdominal examination.

## 3 Investigations

Contrast-enhanced laryngotracheal computed tomography with three-dimensional reconstruction demonstrated tracheal stenosis at the C7–T1 level, with marked luminal narrowing. The narrowest airway diameter was approximately 2 mm, and the stenotic segment measured approximately 2 cm in length (Figures 1A,B,D,E).

**Figure 1:**
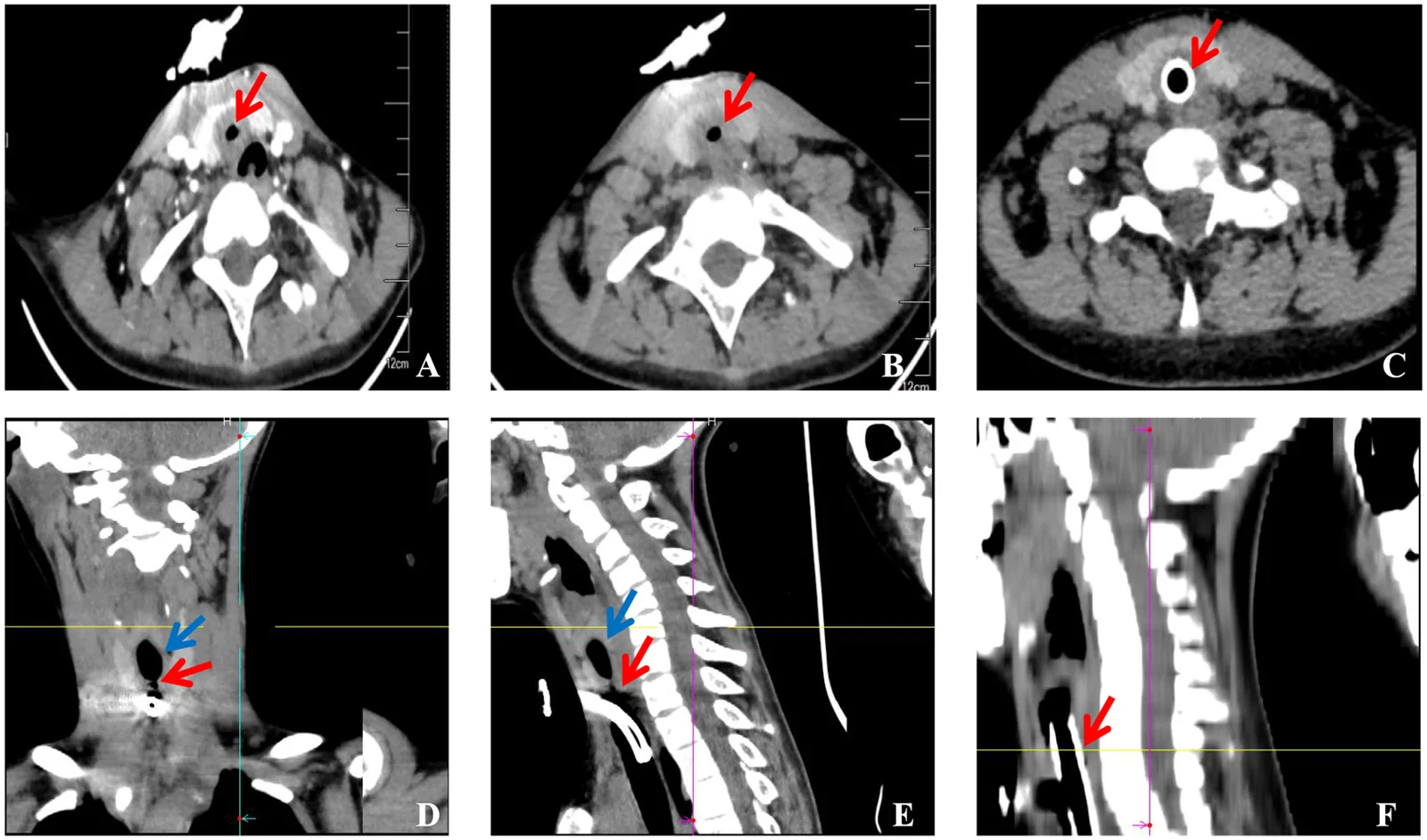
Contrast-enhanced laryngotracheal computed tomography with three-dimensional reconstruction. **(A)** Contrast-enhanced axial CT image (lung window) showing the subglottic stenotic segment (red arrow). **(B)** Non-contrast axial CT image (lung window) showing the subglottic stenotic segment (red arrow). **(C)** Non-contrast axial CT image (lung window) obtained after Montgomery T-tube placement, showing the subglottic airway (red arrow). **(D)** Coronal CT image showing the subglottic stenotic segment (red arrow). The minimum airway diameter was 2 mm and the stenotic segment measured 2 cm in length. The blue arrow indicates the maximum tracheal diameter (13 mm). **(E)** Sagittal CT image showing the subglottic stenotic segment (red arrow). The minimum airway diameter was 2 mm and the stenotic segment measured 2 cm in length. The blue arrow indicates the maximum tracheal diameter (13 mm). **(F)** Sagittal CT image obtained after Montgomery T-tube placement, showing the subglottic airway (red arrow).

Bronchoscopic examination showed posterior distortion of the subglottic airway, with funnel-shaped luminal narrowing. Membranous granulation tissue was present at the base of the stenosis, producing near-complete airway obstruction (Figure 2A).

**Figure 2:**
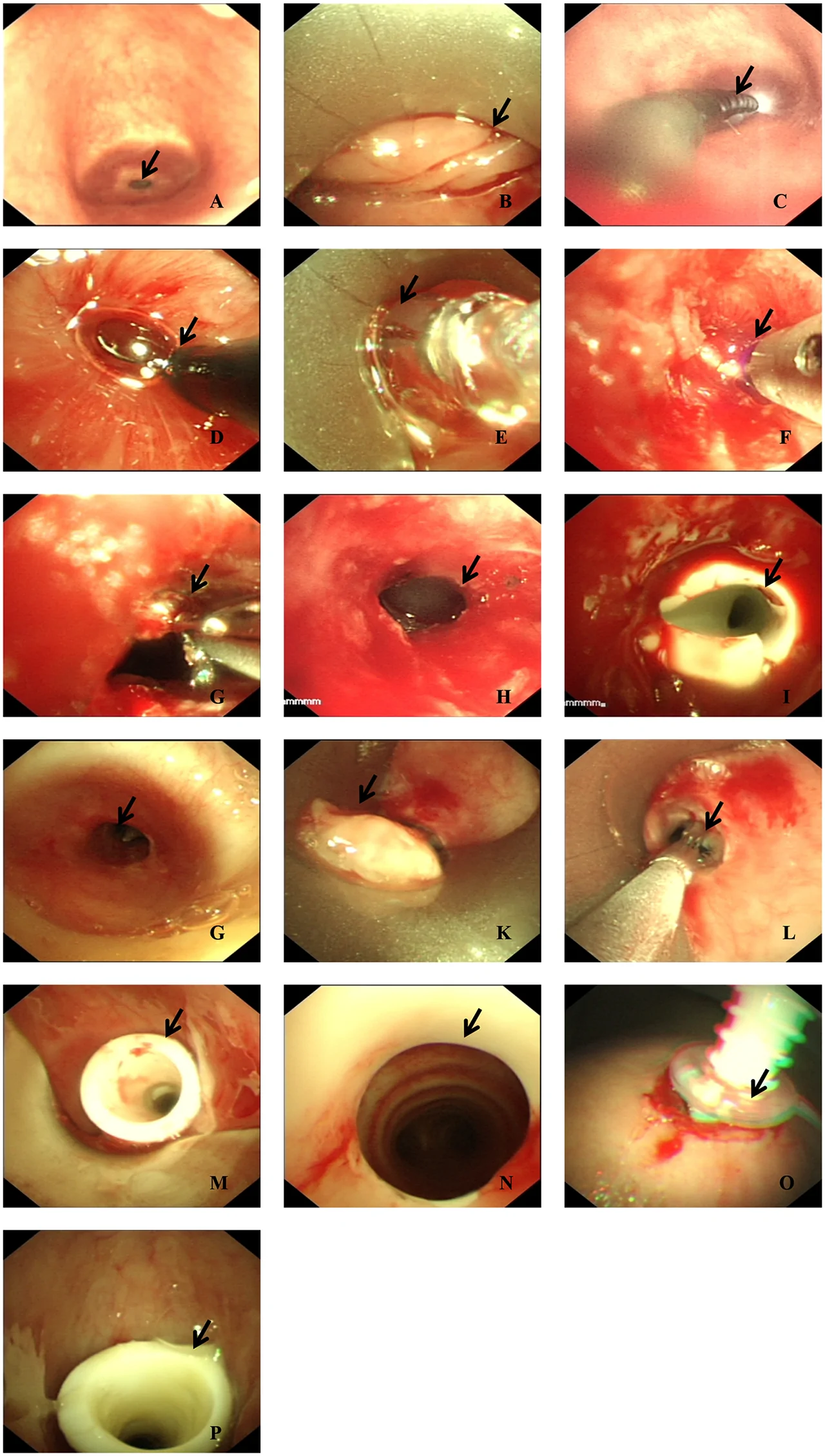
Bronchoscopic findings. **(A)** Bronchoscopic view on April 20, 2026, showing severe subglottic stenosis with posterior airway distortion, funnel-shaped narrowing, and membranous granulation tissue at the distal end of the stenosis, producing near-complete obstruction (approximately 98%, black arrow). **(B)** Introduction of an 11.5-mm rigid bronchoscope on May 13, 2026 (black arrow). **(C)** Advancement of an ultrathin bronchoscope into the stenotic segment for evaluation and guidance (black arrow). **(D)** Electrocautery incision using a needle-knife in cutting mode to release the stenotic segment by circumferential radial incisions (black arrow). **(E)** Sequential balloon dilation using a 6-mm balloon (3 atm for 30 s), followed by an 8-mm balloon (3 atm for 30 s, repeated four times) (black arrow). **(F)** Contact laser therapy (7–10 W, 0.5 s per application, four applications) for ablation of residual tissue (black arrow). **(G)** Removal of necrotic tissue with biopsy forceps (black arrow). **(H)** Airway status after recanalization, with the lumen expanded to approximately 10 mm (black arrow). **(I)** Initial attempt at Montgomery T-tube placement, showing poor expansion of the upper limb (black arrow). **(J)** Bronchoscopic reassessment on May 15, 2026, showing stable mucosa without granulation tissue, necrosis, or restenosis (black arrow). **(K)** Introduction of an 11.5-mm rigid bronchoscope during definitive T-tube placement on May 19, 2026 (black arrow). **(L)** Delivery of the T-tube into the airway using forceps grasping the upper limb (black arrow). **(M)** Upper limb of the Montgomery T-tube *in situ* (black arrow). **(N)** Lower limb of the Montgomery T-tube *in situ* (black arrow). **(O)** External limb of the Montgomery T-tube *in situ* (black arrow). **(P)** Bronchoscopic view of the upper limb of the T-tube on May 22, 2026 (black arrow).

Airway assessment confirmed structural subglottic stenosis characterized by cicatricial contracture and web-like formation. The minimum airway diameter was 2 mm, corresponding to approximately 98% luminal obstruction. The stenotic segment measured 2 cm in length and was classified as Grade III according to the Myer-Cotton grading system.

## 4 Diagnosis

Based on the patient's clinical history, imaging findings, and bronchoscopic evaluation, a diagnosis of Grade III post-intubation subglottic stenosis was established. Additional diagnoses included traumatic subarachnoid hemorrhage (recovery phase) and traumatic cerebral infarction (recovery phase). The patient had near-complete airway obstruction caused by severe post-intubation subglottic stenosis, with aphonia and impaired phonatory function.

## 5 Treatment

Given the severity and complexity of the airway lesion, a multidisciplinary team involving respiratory and critical care medicine, otolaryngology-head and neck surgery, anesthesiology, and interventional endoscopy developed an individualized treatment strategy.

Bronchoscopy revealed marked airway mucosal congestion and edema with abundant purulent secretions, consistent with active airway inflammation. The otolaryngology-head and neck surgery team recommended preoperative airway optimization, including anti-infective and anti-inflammatory therapy, airway humidification, and airway clearance measures. Once airway inflammation was adequately controlled, revision of the tracheostomy and replacement of the metal tracheostomy tube with a size 6.5 plastic tracheostomy tube were planned.

CT and bronchoscopy showed a 2-cm stenotic segment with a minimum diameter of 2 mm, a maximum adjacent airway diameter of approximately 13 mm, and focal cartilaginous damage. Open laryngotracheal reconstruction or cricotracheal resection was considered, but the team favored endoscopic recanalization with temporary T-tube support because the lesion was localized, a residual lumen remained, airway access through the tracheostomy could be maintained, and a less invasive staged approach allowed reassessment before definitive reconstruction. A 10-mm Montgomery T-tube and a size 6.5 plastic tracheostomy tube were selected according to airway dimensions and body size.

Because the upper airway was nearly obstructed, the anesthesiology team planned ventilation through the tracheostomy stoma using a size 4 endotracheal tube after removal of the metal tube. The respiratory team planned electrocautery incision, laser ablation, and balloon dilation, followed by T-tube placement after reassessment of the airway mucosa.

Preoperative airway optimization consisted of a 2-week course of intravenous ticarcillin-clavulanate (1.6 g every 8 h), oral cetirizine (once daily), intravenous methylprednisolone (20 mg once daily), mometasone furoate nasal spray (two sprays twice daily), ultrasonic airway humidification twice daily, and cough training. After treatment, the patient was able to tolerate complete tracheostomy tube occlusion for 10-20 min with clear phonation, suggesting significant improvement in airway inflammation.

On April 30, 2026, the metal tracheostomy tube was replaced with a size 6.5 plastic tracheostomy tube. After removal of the size 6 metal tube, a size 4 endotracheal tube was inserted through the tracheostomy stoma and connected to invasive mechanical ventilation (Figure 3, red arrow). Tracheostomy revision was then performed, followed by placement of a size 6.5 plastic tracheostomy tube (Henan Tuoren Medical Device Group Co., Ltd., Henan, China).

**Figure 3:**
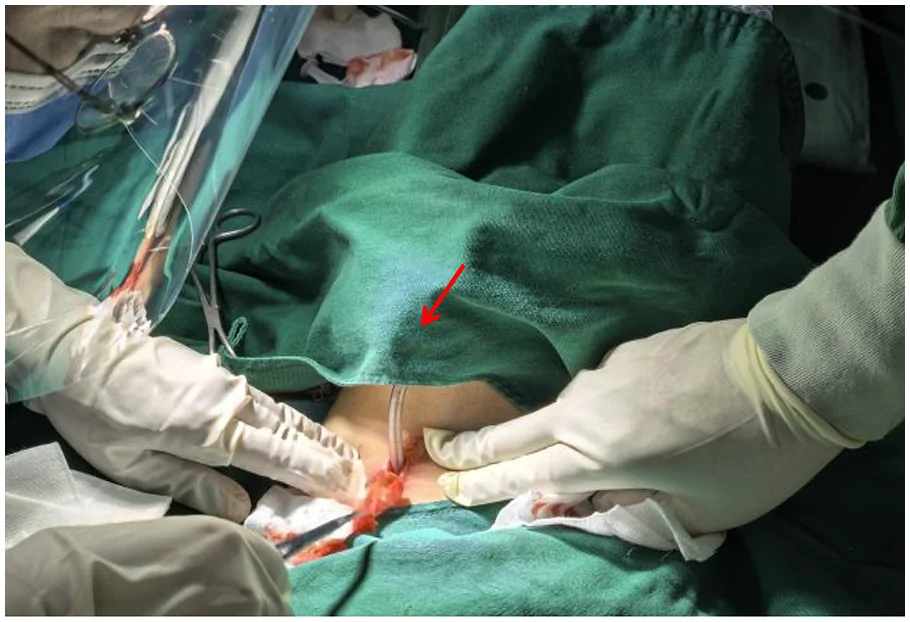
A size 4 endotracheal tube inserted through the tracheostomy stoma and connected to invasive mechanical ventilation (red arrow).

On the same day, airway recanalization was performed under general anesthesia. The tracheostomy tube was connected to invasive mechanical ventilation using volume-controlled ventilation with a fraction of inspired oxygen of 100%, respiratory rate of 30 breaths/min, tidal volume of 300 mL, and positive end-expiratory pressure of 0 cm H₂O.

An 11.5-mm rigid bronchoscope (Xi'an Dunbo Medical Instrument Co., Ltd., Xi'an, China) was introduced to maintain airway access (Figure 2B), and an ultrathin bronchoscope (Shenzhen Insight Medical Technology Co., Ltd., Shenzhen, China) was advanced into the stenotic segment for guidance (Figure 2C). Electrocautery incision was performed using a needle-knife electrode at 35–45 W for 2–3 s per application, with four applications in total, creating circumferential radial incisions in the scar tissue (Figure 2D).

Sequential balloon dilation was then performed using a 6-mm balloon catheter at 3 atm for 30 s, followed by an 8-mm balloon catheter at 3 atm for 30 s per dilation, repeated four times (Figure 2E). Residual scar tissue was treated using contact laser therapy at 7–10 W for 0.5 s per application, with four applications in total (Figure 2F). Necrotic tissue was repeatedly removed using biopsy forceps (Figure 2G). These interventions expanded the airway lumen from approximately 2 mm before treatment (Figure 2A) to 10 mm after recanalization (Figure 2H).

After airway recanalization, the distance from the superior margin of the tracheostomy stoma to the upper border of the stenotic segment was measured. The upper limb of the Montgomery T-tube (Boston Medical Products Inc., Shrewsbury, MA, United States) was trimmed to 25 mm, and the lower limb was trimmed to approximately 30 mm because the distal airway remained patent.

The initial attempt to place the 10-mm Montgomery T-tube failed: airway curvature, mucosal bleeding, and edema prevented full expansion of the upper limb (Figure 2I). The tube was temporarily removed.

Bronchoscopic reassessment two days later demonstrated resolution of mucosal edema and congestion without significant granulation tissue, necrotic tissue formation, or restenosis (Figure 2J).

After stabilization of the airway mucosa, definitive Montgomery T-tube placement was performed on May 19, 2026, under the guidance of an 11.5-mm rigid bronchoscope. The tracheostomy tube was removed, the stoma was disinfected with povidone-iodine, and a traction suture was passed through the T-tube. The device was introduced through the tracheostomy stoma using rigid forceps, and the traction suture was pulled upward until full expansion of the upper limb was achieved. Bronchoscopic examination confirmed satisfactory positioning of the T-tube, with a patent lumen spanning the stenotic segment above the tracheostomy site (Figures 2K,L).

The final configuration consisted of a 2.5-cm upper limb located within the subglottic airway (Figure 2M) and a 3.0-cm lower limb positioned above the carina (Figure 2N).

## 6 Outcome and follow-up

Postoperatively, the patient received anti-infective and anti-inflammatory treatment, nebulized ambroxol and budesonide, airway humidification, and cough training.

Laryngotracheal CT 1 day after T-tube placement showed improved airway patency (Figures 1C,F).

Bronchoscopy 3 days after implantation showed the upper limb approximately 0.5 cm below the glottis. The T-tube was well positioned and patent, without secretion retention or granulation tissue; phonation was restored (Figure 2P). The clinical timeline and objective airway outcomes are summarized in Table 1.

The external limb was kept capped when tolerated, with regular humidification and airway clearance. Bronchoscopic surveillance was planned at 6 and 12 weeks and every 3 months thereafter to assess tube position, granulation tissue, secretion retention, and restenosis.

## 7 Discussion

Acquired SGS most often follows airway instrumentation. Mechanical compression and mucosal ischemia after intubation can trigger inflammation, fibrosis, and scar contracture, producing fixed airway narrowing (1). In this patient, prolonged intubation and subsequent tracheostomy provided a plausible clinical context for the severe cicatricial SGS (3).

The diagnosis of SGS relies on clinical history, symptoms, imaging studies, and endoscopic assessment; among these, endoscopy is the primary tool for evaluating the location, length, morphology, and severity of the stenotic lesion. In this case, neither laryngoscopy nor laryngotracheal computed tomography fully demonstrated the severity of the subglottic lesion. Bronchoscopy, however, clearly identified near-complete obstruction of the subglottic airway. The stenotic segment exhibited posterior distortion, funnel-shaped narrowing, and membranous granulation tissue formation, resulting in approximately 98% luminal obstruction over a 2-cm segment. According to the Myer-Cotton classification, the lesion was categorized as Grade III SGS. In patients with failed tracheostomy occlusion, difficult decannulation, stridor, or unexplained dyspnea, direct endoscopic assessment is therefore important for defining lesion morphology and procedural risk (5, 8).

Treatment selection in pediatric and adolescent SGS depends on stenosis grade, length and location, airway framework involvement, tracheomalacia, previous interventions, comorbidities, and the feasibility of maintaining ventilation (4, 9). Endoscopic dilation or scar incision is generally favored for selected short or less mature lesions, whereas laryngotracheal reconstruction or cricotracheal resection is considered for advanced, recurrent, framework-compromising, or complex stenosis (10, 11). Recent reports also emphasize individualized selection when cicatricial stenosis coexists with tracheomalacia or extensive airway damage (12, 13).

For this patient, the 2-cm localized stenosis, residual lumen, established tracheostomy access, and need to minimize procedural trauma supported a staged endoscopic approach. The aim was not to replace open reconstruction, but to restore a stable lumen while preserving the option of later surgery if restenosis or airway instability developed.

Airway optimization preceded intervention because mucosal inflammation and secretions can impair visualization and increase procedural difficulty (14). Recanalization combined radial electrocautery incision, balloon dilation, and limited laser ablation, enlarging the lumen from 2 to 10 mm. The first T-tube attempt failed because edema and bleeding prevented upper-limb expansion; reassessment after mucosal stabilization allowed successful delayed implantation. This sequence suggests that immediate stenting should be reconsidered when the recanalized airway remains markedly inflamed.

Montgomery T-tube can maintain patency and permit phonation, but their long-term use carries risks of granulation tissue, mucus retention or crusting, migration, infection, and recurrent obstruction (15–17). Granulation tends to occur near tube margins and may be promoted by friction or an upper limb positioned too close to the glottis. Appropriate sizing, adequate distance from the vocal folds, humidification, airway clearance, caregiver education, and scheduled bronchoscopy are therefore central to prevention and early management.

Delayed restenosis remains a concern even after an initially favorable result, particularly in growing children and in patients with unstable airway framework (13). Follow-up should document endoscopic caliber, tube position, respiratory symptoms, voice, swallowing, and eventual decannulation status. In this case, early CT and bronchoscopy confirmed patency and restored phonation, but the available follow-up is short. Long-term durability, late restenosis, and eventual T-tube removal or decannulation cannot yet be determined; this is the main limitation of the report.

A practical treatment framework is summarized in Table 2. It is intended as a decision aid rather than a fixed algorithm because pediatric airway reconstruction remains highly dependent on anatomy, airway stability, comorbidities, and local expertise.

**Table 2:** Practical treatment framework for pediatric/adolescent subglottic stenosis.

Approach	Typical setting	Main limitation	Role in this case
Endoscopic dilation/incision	Selected short lesions with a residual lumen and secure airway access	Recurrence; repeat procedures	Initial recanalization
Montgomery T-tube	Temporary internal support after recanalization	Granulation, secretion retention, migration or blockage	Airway support and phonation
Laryngotracheal reconstruction	Moderate-to-severe or complex stenosis requiring airway expansion	Open surgery; graft/stent morbidity	Alternative option
Cricotracheal resection	Severe, mature, recurrent, or framework-compromising short stenosis	Technically demanding; anastomotic risk	If endoscopic treatment fails or instability develops

## 8 Conclusion

This case illustrates several considerations in the management of severe post-intubation SGS in adolescents. SGS should be suspected in patients with a history of traumatic brain injury, intensive care unit admission, endotracheal intubation, mechanical ventilation, or tracheostomy who subsequently develop failed tracheostomy tube occlusion, difficult decannulation, stridor, or unexplained dyspnea, rather than attributing these symptoms solely to pulmonary infection, airway spasm, or upper airway collapse (2, 3).

The management of severe SGS requires a multidisciplinary approach involving interventional pulmonology, otolaryngology-head and neck surgery, anesthesiology, critical care, rehabilitation, and nursing teams. Potential risks, including loss of airway, bleeding, perforation, postoperative edema, and mucus plugging, should be carefully anticipated before intervention (8, 17).

Montgomery T-tube placement is an important therapeutic option for complex high-grade benign airway stenosis. Successful outcomes depend on appropriate tube sizing, accurate placement, adequate control of airway inflammation, and long-term endoscopic follow-up (18, 19).

Outcome assessment in pediatric and adolescent patients differs from that in adults. In younger patients, greater emphasis should be placed on growth and development, restoration of phonatory and swallowing functions, and recovery of psychosocial communication abilities; in adults, management of comorbidities, prevention of restenosis, and long-term quality of life tend to be the primary concerns (20–22).

## Patient consent

Written informed consent was obtained from the legal guardian for the publication of this case report and any accompanying images. The guardian understands that all identifying information has been anonymized.

## Data Availability

No datasets were generated or analyzed during the current study. Patient-related information cannot be publicly shared due to privacy and confidentiality restrictions.
